# PD-L1 is a critical mediator of regulatory B cells and T cells in invasive breast cancer

**DOI:** 10.1038/srep35651

**Published:** 2016-10-20

**Authors:** Honggeng Guan, Yuqiu Wan, Jing Lan, Qin Wang, Zhangyu Wang, Yecheng Li, Jiqing Zheng, Xueguang Zhang, Zemin Wang, Yueping Shen, Fang Xie

**Affiliations:** 1 The Department of Pathology, Medical College of Soochow University, 199 Renai Road, Suzhou, 215123, P. R. China; 2 Department of General Surgery, The First Affiliated Hospital of Soochow University, 188 Shizi Street, Suzhou, 215006, P. R. China; 3 Department of Immunology, Medical College of Soochow University, 199 Renai Road, Suzhou, 215123, P. R. China; 4 Department of General Surgery, The Second Affiliated Hospital of Soochow University, 1055 Sanxiang Road, Suzhou 215004, P. R. China; 5 Clinical Immunology Institute, The First Affiliated Hospital of Soochow University, 188 Shizi Street, Suzhou, 215006, P. R. China; 6 Department of Environmental Health, School of Public Health, Indiana University, Bloomington, IN 47405, USA; 7 The Department of Epidemiology and Biostatistics, School of Public Health, Soochow University, 199 Renai Road, Suzhou 215123, P. R. China

## Abstract

Regulatory T cells (Tregs), a key mediator in regulating anti-tumor immune suppression, tumor immune escape, metastasis and relapse, are considered an important therapeutic target in immunotherapy of human cancers. In the present investigation, elevated CD19^+^ CD24^+^ CD38^+^ regulatory B cells (Bregs) were observed in PBMCs of invasive carcinoma of breast (IBCa) patients compared with that in patients with fibroadenoma (FIBma) or healthy individuals, and the positive correlation existed between Bregs and CD4^+^ CD25^+^ CD127^−^ Tregs (*r* = 0.316, *P* = 0.001). We found that PD-L1 expression was higher on Bregs in IBCa patients compared with patients with FIBma or healthy individuals (*P* < 0.05, respectively), and that a tight correlation exists between CD19^+^ CD24^+^ CD38^+^ PD-L1^+^ Bregs and CD19^+^ CD24^+^ CD38^+^ Bregs (*r* = 0.267, *P* = 0.007), poor TNM phases and up-regulated expression of PD-L1 on Bregs. The pattern of PD-1 expression on CD4^+^ T cells indicated that high level of PD-1^hi^ expressed on CD4^+^ CD25^+^ CD127^+^ effector T cells (*P* < 0.001). More importantly, the presence of PD-L1 on Bregs was positively correlated with Tregs (*r* = 0.299, *P* = 0.003), but negatively correlated with PD-1^hi^ effector T cells (*r* = −0.22, *P* = 0.031). Together, results of the present study indicated that PD-L1 is an important molecule on Bregs, mediated the generation of Tregs in IBCa.

Regulatory B cells (Bregs), a subset of B cells, play a suppressive role in autoimmune diseases, inflammation, and anti-tumor immune response^1^,^2^,^3^,^4^,^5^. Based on the expression of various surface molecules, several populations of Bregs have been reported including B10 cells^6^, CD1d^hi^ CD5^+^ CD19^+^ B cells^7^, CD19^+^ CD24^+^ CD38^+^ B cells^2^ or CD19^+^ CD24^+^ CD27^+^ B cells^8^ and so on. Recently, IL-10-producing was recognized as one of the most important characters of functional Bregs^6^,^9^,^10^,^11^. Increasing evidence showed that, IL-10, as an inhibitory cytokine, suppresses the differentiation and proliferation of Th17, inhibits the secretion of IFN-γ, and reduces the accumulation of NK cells^5^. In one of our early studies, we also found high percentage and density of CD19^+^ B cells in the tissues of invasive carcinoma of breast (IBCa) which expressed IL-10 in cytoplasm. We demonstrated that CD19^+^ B cells from IBCa patients but not that from healthy individuals could induce the expansion of Treg cells *in vitro*^12^. The immunosuppressive effects of Bregs have also been shown to be mediated via by promoting Tregs in mice models of autoimmune diseases^13^, and non-autoimmune diseases including cancers^14^. Nevertheless, the function and mechanism of Bregs in immune response remain poorly understood.

Programmed death-ligand 1 (PD-L1), as a critical suppressive molecule, constitutively expresses on B lymphocytes, T lymphocytes, dendritic cells and monocytes^15^,^16^,^17^. The expression of PD-L1 is induced by ligation of cell surface receptors and/or stimulation with the T_H_1-associated cytokine IFNγ^15^. Its receptor, the programmed death-1 (PD-1), is up-regulated in activated T or B cells^17^. The PD-1/PD-L1 signaling axis has been shown to be a critical regulator for maintaining peripheral tolerance^15^.

It is clear that there was an increased number of CD19^+^ CD24^+^ CD38^+^ Bregs in the peripheral blood mononuclear cells (PBMCs) of IBCa patients^12^, however it is not currently known whether PD-1/PD-L1 expressed on CD19^+^ CD24^+^ CD38^+^ Bregs acts exclusively on Tregs or other components of IBCa. In the present study, in an effort to further understand the role of Bregs in the etiology and pathogenesis of breast cancer, we examined CD4^+^ T cells, CD19^+^ B cells and their subsets in PBMCs of breast tumor patients, and investigated the relationship among CD4^+^ T cells, CD19^+^ B cells and their subsets in breast tumor.

## Results

### CD4^+^CD25^+^CD127^low/−^ Tregs predominated in PBMCs of IBCa patients

**Figure 1:**
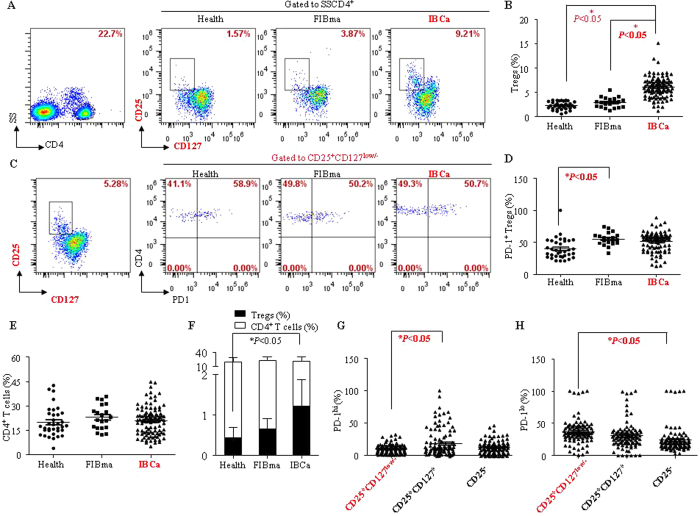
CD4^+^CD25^+^CD127^low/−^ T lymphocyte predominated in PBMCs of IBCa patients. To investigate the phenotype and subsets of CD4^+^ T cells in patients and healthy individuals, PBMCs were isolated from peripheral blood, incubated with CD4-ECD, CD25-PE-cy7, CD127-FITC or PD-1-PE, and analyzed by flow cytometry. (**A**,**B**) The percentage of CD4^+^CD25^+^CD127^low/−^ T cells was among the highest in PBMCs of IBCa patients (6.06 ± 2.17%) compared with FIBma patients (2.91 ± 1.06%) and health individuals (2.28 ± 0.79%) (*P* < 0.05 for all). (**C**,**D**) The expression of PD-1 on CD4^+^CD25^+^CD127^low/−^ Tregs in PBMCs of health individuals, FIBma and IBCa patients analyzed by flow cytometry. These was significant difference between the PBMCs of FIBma patients and health individuals (*P* < 0.05). (**E**) The percentages of CD4^**+**^ T cells in PBMCs of health individuals, FIBma and IBCa patients were 19.90 ± 9.02%, 22.98 ± 7.51%, and 20.74 ± 8.84%, respectively. And there no difference was found between health individuals and FIBma patients or health individuals and IBCa patients (*P* > 0.05). (**F**) The ratio of CD4^+^CD25^+^CD127^low/−^ Tregs and CD4^**+**^ T cells was highest in PBMCs of IBCa patients compared with health individuals and FIBma (*P* < 0.001). (**G**,**H**) The expression of PD-1 compartmentalized into two distinct (PD-1^lo^ and PD-1^hi^) populations. The high level PD-1^hi^ expression was found on CD4^+^CD25^+^CD127^+^ T cells (20.00 ± 22.15%) and CD4^+^CD25^−^ T cells (13.92 ± 10.15%), compared with CD4^+^CD25^+^CD127^low/−^ Tregs (9.57 ± 7.27%). And there was significantly difference between CD4^+^CD25^+^CD127^low/−^ Tregs and CD4^+^CD25^+^CD127^+^ effector T cells (*P* < 0.05). On the other hand, there was high level PD-1^lo^ expression was found on CD4^+^CD25^+^CD127^low/−^ Tregs (37.17 ± 19.27%), compared with CD4^+^CD25^+^CD127^+^ effector T cells (31.03 ± 20.84%) or CD4^+^CD25^−^ T cells (23.87 ± 19.68%) (*P* > 0.05 and *P* < 0.05, respectively).

CD4^**+**^ T lymphocytes, regarded as T helper cells, regulate the immune responses by activating other immune cells or dividing to Tregs to suppress immune reaction. In the present study, we evaluated the percentages of CD4^**+**^ T cells and its subsets in the PBMCs of breast tumor patients. The percentages of CD4^**+**^ T cells in PBMCs of health individuals (19.90 ± 9.02%), fibroadenoma (FIBma) (22.98 ± 7.51%) and IBCa patients (20.74 ± 8.84%) were comparable (*P* > 0.05; Fig. 1A,E). However, The percentage of CD4^+^CD25^+^CD127^low/−^ T cells was significantly higher in the PBMCs of IBCa patients (6.06 ± 2.17%) compared with that in FIBma patients (2.91 ± 1.06%) and health individuals (2.28 ± 0.79%) (*P* < 0.05 for both; Fig. 1A,B). Similarly, the ratio of CD4^+^CD25^+^CD127^low/−^ Tregs and CD4^**+**^ T cells was among the highest in IBCa patients in comparison with health individuals and FIBma patients (*P* < 0.05; Fig. 1F).

Because PD-1 is an important molecule in immune suppression, we then evaluated the expression of PD-1 on CD4^+^ T cells and its subsets including CD4^+^CD25^+^CD127^low/−^ Tregs in PBMCs. Based on the the expression level of PD-1, each cell population was divided into two distinct subpopulations, PD-1^lo^ and PD-1^hi^ (Fig. S1). Although the expression levels of PD-1 on CD4^+^CD25^+^CD127^low/−^ Tregs were significantly different between the PBMCs of FIBma (55.19 ± 9.98%) and healthy individuals (39.95 ± 16.10%)(*P* < 0.05; Fig. 1C,D), no significant difference between IBCa patients (48.10 ± 18.60%) and healthy individuals (*P* > 0.05; Fig. 1C,D) were seen. However, the percentage of PD-1^hi^ CD4^+^CD25^+^CD127^low/−^ Tregs (9.57 ± 7.27%) were significantly lower than the PD-1^hi^ CD4^+^CD25^+^CD127^+^ T cells (20.00 ± 22.15%) in IBCa (*P* < 0.05; Fig. 1G). The percentage of PD-1^hi^ CD4^+^CD25^−^ T cells was 13.92 ± 10.15%, which was not significantly different from PD-1^hi^ CD4^+^CD25^+^CD127^low/−^ Tregs or PD-1^hi^ CD4^+^CD25^+^CD127^+^ effector T cells (Fig. 1G). In contrast, there were statistically significantly more PD-1^lo^ CD4^+^CD25^+^CD127^low/−^ Tregs (37.17 ± 19.27%) than PD-1^lo^ CD4^+^CD25^+^CD127^+^ effector T cells (31.03 ± 20.84%) and PD-1^lo^ CD4^+^CD25^−^ T (23.87 ± 19.68%) (*P* > 0.05 and *P* < 0.05, respectively) in IBCa (Fig. 1H).

Further analysis of the relationship among the percentages of CD4^+^ T cells, CD4^+^ CD25^+^ CD127^low/−^ Tregs, and CD4^+^ CD25^+^ CD127^low/−^ PD-1^+^ Tregs in IBCa and the histopathological characteristics of IBCa revealed that the CD4^+^ T cells were higher in patients of 49 years or older (*P* = 0.047) (Table 1). The percentage of the above cell populations were not significantly different among all other histopathological characters examined including tumor grade, status of metastasis, TNM staging, ER, PR and HER2 status, as well as tumor size (Table 1).

### IL-10^+^ B cells were enriched in the CD19^+^CD24^+^CD38^+^ B cell population

**Figure 2:**
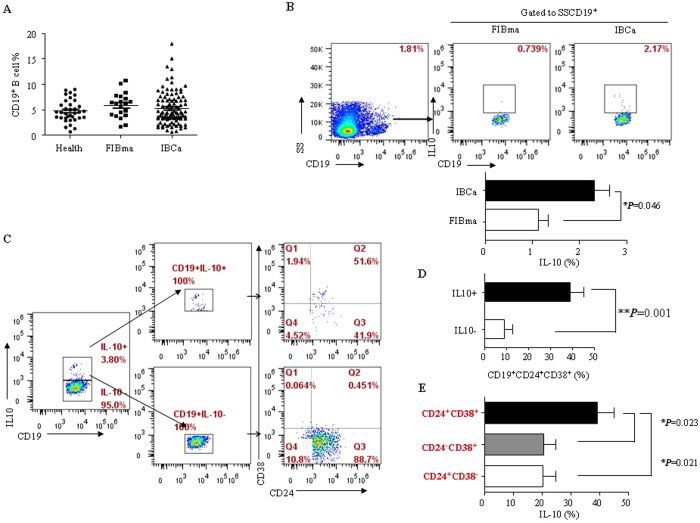
IL-10^+^ B cell were enriched with the CD19^+^CD24^+^CD38^+^ B cells population. (**A**) The percentages of CD19^+^ B cells in PBMCs had no different among FIBma patients, IBCa patients and health individuals (5.89 ± 2.44%, 5.31 ± 3.23%, and 4.75 ± 2.16%, respectively). (**B**) A higher level of IL-10 (2.31 ± 1.01%) in IBCa patients compared with FIBma patients (1.12 ± 0.41%) (*P* = 0.046). (**C**,**D**) In our early report, CD19^+^CD24^+^CD38^+^ B cell was identified as regulatory B cells in breast cancer^12^. So, to identify the phenotype of CD19^+^L-10^+^ B cells in PBMCs of IBCa, stained with CD24 and CD38 and analyzed by flow cytometry. A significantly higher percentage of CD24^+^CD38^+^ in the CD19^+^IL-10^+^ group (39.18 ± 5.98%) compared to that in the CD19^+^IL-10^−^ group (8.87 ± 4.08%) (*P* = 0.001). (**E**) The higher level of IL-10 secreted by CD19^+^CD24^+^CD38^+^ B lymphocytes (39.18 ± 18.91%) than CD19^+^CD24^−^CD38^+^ B cells (20.72 ± 13.01%) or CD19^+^CD24^+^CD38^−^ B cells (20.48 ± 13.46%) (*P* = 0.023, *P* = 0.0231).

Different from the positive regulation in immune response, the new functions of B cells in antitumor immunity warrant further investigation. CD19^+^IL-10^+^ B cells, also known as B10, were regarded as regulatory B cells which play an important role in immune suppression. In present study, we evaluated the percentage of CD19^+^ B cells in PBMCs of breast tumor patients. No significant difference was found in the percentage of CD19^+^ B cells in PBMCs among FIBma, IBCa patients and healthy individuals (5.89 ± 2.44%, 5.31 ± 3.23%, and 4.75 ± 2.16%, respectively) (Fig. 2A). However, the level of IL-10 secreting CD19^+^ B cells was higher in IBCa patients compared with that in FIBma patients (2.31 ± 1.01% vs 1.12 ± 0.41%; *P* = 0.046) (Fig. 2B).

We have previously demonstrated that CD19^+^CD24^+^CD38^+^ B cells were regulatory B cells in breast cancer^12^. In the current study, to further determine the phenotype of CD19^+^IL-10^+^ B cells in PBMCs of IBCa, isolated CD19^+^IL-10^+^ B cells were labeled with anti-CD24 and anti-CD38 antibodies and analyzed by flow cytometry. A significantly higher percentage of CD24^+^CD38^+^ in the CD19^+^IL-10^+^ group (39.18 ± 5.98%) was seen compared to that in the CD19^+^IL-10^−^ group (8.87 ± 4.08%) (*P* = 0.001) (Fig. 2C,D). Consistent with the findings of our previous study^12^, the level of IL-10 was significantly higher in the CD19^+^CD24^+^CD38^+^ B lymphocytes (39.18 ± 18.91%) than in the CD19^+^CD24^−^CD38^+^ B cells (20.72 ± 13.01%) and CD19^+^CD24^+^CD38^−^ B cells (20.48 ± 13.46%) (*P* = 0.023 and *P* = 0.0231, respectively) (Fig. 2E).

### CD19^+^CD24^+^CD38^+^ Bregs and its subsets were expanded in breast cancer patients

**Figure 3:**
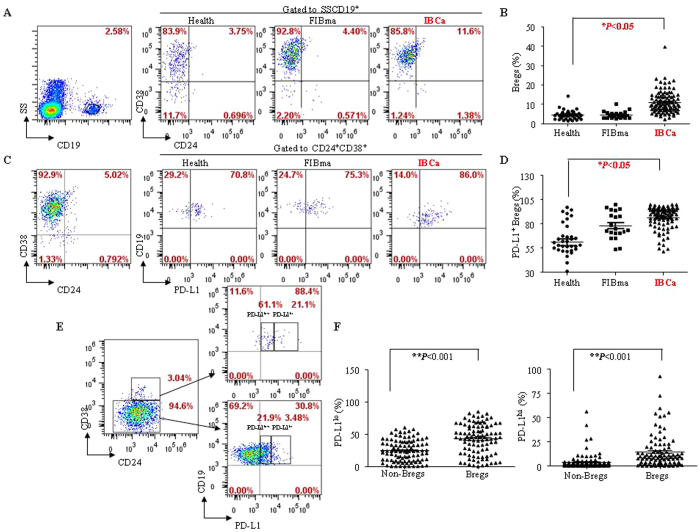
CD19^+^CD24^+^CD38^+^ B cell and its subsets are expanded in breast cancer patients. (**A**,**B**) The percentage of CD19^+^CD24^+^CD38^+^ B cells in PBMCs of IBCa patients (10.99 ± 5.70%) was significant higher than in that of FIBma patients (4.48 ± 1.91%) and health individuals (4.49 ± 2.48%) (*P* < 0.05). (**C**,**D**) PD-L1 was detected on CD19^+^CD24^+^CD38^+^ Bregs of breast tumor patients and health individuals. The significant higher CD19^+^CD24^+^CD38^+^PD-L1^+^ subset (83.74 ± 19.32%) was observed in PBMCs of IBCa patients compared with that in FIBma patients (78.40 ± 13.96%) or health individuals (61.13 ± 20.51%) (*P* < 0.05). (**E**,**F**) The pattern of PD-L1 on CD19^+^CD24^+^CD38^+^ Bregs in PBMCs of IBCa patients was analyzed by cytometry. Wherever PD-L1^lo^ or PD-L1^hi^ expression was significant higher on CD19^+^CD24^+^CD38^+^ Bregs (42.36 ± 23.27% or 14.02 ± 16.99%) than that on Non-Bregs (24.58 ± 16.51% or 3.92 ± 8.18%) in IBCa (*P* < 0.001 for all).

Subsequently, we investigated the distribution of CD19^+^CD24^+^CD38^+^ Bregs and various subsets in the PBMCs of breast tumor patients. In comparison with FIBma patients and healthy participants (4.48 ± 1.91% and 4.49 ± 2.48%, respectively), the percentage of CD19^+^CD24^+^CD38^+^ B cells was significantly higher in the PBMCs of IBCa patients with 10.99 ± 5.70% (*P* < 0.05) (Fig. 3A,B).

Similar to the experiments in CD4^+^ T cells and Tregs, we determined the expression pattern of the negative immune regulator PD-L1 in B cells and CD19^+^CD24^+^CD38^+^ Bregs (Fig. 3C). Significantly higher level of CD19^+^CD24^+^CD38^+^PD-L1^+^ subset (83.74 ± 19.32%) was observed in the PBMCs of IBCa patients compared with that in FIBma patients (78.40 ± 13.96%) or healthy individuals (61.13 ± 20.51%) (*P* < 0.05, respectively) (Fig. 3C,D). In addition, both the PD-L1^lo^ and PD-L1^hi^ expression were found to be significantly higher on CD19^+^CD24^+^CD38^+^ Bregs (42.36 ± 23.27% and 14.02 ± 16.99%) compared with non-Bregs (24.58 ± 16.51% and 3.92 ± 8.18%; *P* < 0.001 for both) (Fig. 3E,F).

We further investigated the relationships among the levels of CD19^+^ B cells, CD19^+^ CD24^+^ CD38^+^ Bregs, as well as CD19^+^ CD24^+^ CD38^+^ PD-L1^+^ Bregs in IBCa and the histopathological characteristics of IBCa, and found that the CD19^+^ B cells were significantly higher in patients with tumors more than 3cm (*P* = 0.019) and CD19^+^ CD24^+^ CD38^+^ PD-L1^+^ Bregs were among the highest patient at TNM stage T4 (*P* = 0.011) (Table 2).

### The relationship between CD4^+^ T cell subsets and CD19^+^ B cell subsets in IBCa

**Figure 4:**
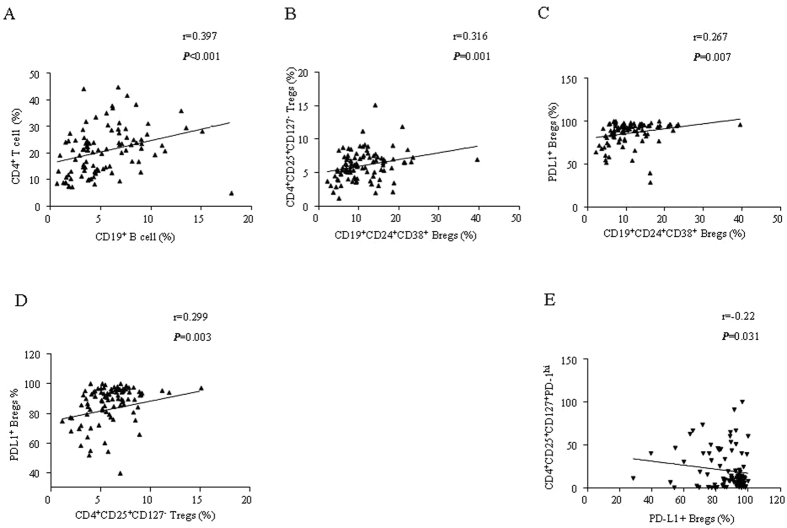
The correlation between CD4^+^ T cell subsets and CD19^+^ B cell subsets in IBCa. (**A**) The positive correlation was found between the percentages of CD4^+^ T cells and CD19^+^ B cells (*r* = 0.397, *P* < 0.001). (**B**) The positive correlation was found between CD4^+^CD25^+^CD127^low/−^ Tregs and CD19^+^CD24^+^CD38^+^ Bregs (*r* = 0.316, *P* = 0.001). (**C**) The positive correlation was found between PD-L1^+^ Bregs and Bregs (*r* = 0.267, *P* = 0.007). (**D**) The positive correlation was found between PD-L1^+^ Bregs and CD4^+^CD25^+^CD127^low/−^ Tregs (*r* = 0.299, *P* = 0.003). (**E**) There was significantly negative correlation between PD-L1^+^ Bregs vs PD-1^hi^ CD4^+^ CD25^+^ CD127^+^ effector T cells (*r* = −0.220, *P* = 0.031).

In our previous studies, we have shown that B cells from IBCa could induce Tregs *in vitro*^12^. In the present study, we further examined the correlation between CD4^+^ T cell subsets and CD19^+^ B cell subsets in PBMCs of IBCa patients. The results showed a positive correlation between the percentages of CD4^+^ T cells and CD19^+^ B cells (*r* = 0.397, *P* < 0.001), CD4^+^CD25^+^CD127^low/−^ Tregs and CD19^+^CD24^+^CD38^+^ Bregs (*r* = 0.316, *P* = 0.001), PD-L1^+^ Bregs and Bregs (*r* = 0.267, *P* = 0.007), PD-L1^+^ Bregs and CD4^+^CD25^+^CD127^low/−^ Tregs (*r* = 0.299, *P* = 0.003) (Fig. 4A–D). In contrast, PD-L1^+^ Bregs and PD-1^hi^ CD4^+^ CD25^+^ CD127^+^ effector T cells were inversely correlated with each other (*r* = -0.220, *P* = 0.031; Fig. 4E).

## Discussion

Although the overall survival rate has been improved considerably over the past few decades with the advancement of treatment modalities, breast cancer is still the leading causes of cancer mortality in women^18^. The immunosuppression and immune escape are regarded as predicting factors for poor prognosis of solid cancers. Regulatory T cells (Tregs), defined as CD4^+^CD25^+^Foxp3^+^ or CD4^+^CD25^+^CD127^low/−^, mediate peripheral tolerance and prevent autoimmunity in healthy individuals. It has been recognized that Tregs serve as a key mediator that regulates and maintains the balance of immune response by suppressing the expansion of effector T cells^19^. Therefore, Tregs play a beneficial role in treatment of autoimmunity diseases. In contrast, there is usually an increased number of Tregs in the peripheral blood or tumor sites in patients who have cancer, including head and neck squamous cell carcinoma, breast cancer, lung cancer, colorectal cancer, pancreatic cancer, ovarian cancer and melanoma^20^,^21^,^22^. Numerous reports showed Tregs were associated with inhibition of immune response against cancer, tumor immune escape and metastasis^19^. Higher number of Tregs has been associated with higher risk of relapse and shorter relapse-free survival. A recent investigation suggested that Tregs may be an important pathological factor predicting a response to hormone therapy or chemotherapy in breast cancer patients and can be potential therapeutic target for breast cancer^23^. However, the mechanisms that regulate Tregs activity is only beginning to be elucidated.

We have shown in the present study and our previous report^12^ that there was a significantly elevated number of CD4^+^ CD25^+^ Foxp3^+^ or CD4^+^ CD25^+^ CD127^−^ Tregs in the PBMCs of IBCa patients compared with that in patients with breast benign tumor or healthy women. We revealed that it was the ratio of CD4^+^ T cells to CD4^+^ CD25^+^ CD127^−^ Treg instead of their number changes that governs their suppressive activity. On the other hand, CD19^+^ B cells from IBCa was recently shown to promote the expansiveness of Tregs in a T and B cell co-culture system, and an elevated number of CD19^+^ CD24^+^ CD38^+^ B cells, identified as Bregs, were found in IBCa^12^. The majority of studies on Bregs have focused on their suppression in the ranges of autoimmune, allergic conditions and cancer in both mice and man. The immune regulatory functions of Tregs have been described, yet little is known about the regulatory role B cells toward on Tregs responses against tumor. In the present investigation, significantly high percentage of CD19^+^ CD24^+^ CD38^+^ Bregs was seen in the PMBCs of IBCa patients compared with that in benign tumor patients or healthy individuals. A positive correlation between CD19^+^ CD24^+^ CD38^+^ Bregs and CD4^+^ CD25^+^ CD127^−^ Tregs was also observed.

We further investigated the function and relationship of PD-L1/PD-1 between Tregs and Bregs. PD-L1 as a ligand of PD-1 is constitutively expressed on B lymophocytes, macrophages and dendritic cells (DCs)^15^. Meanwhile, PD-1 is also inducible on activated T-cell subsets^24^. The PD-1/PD-L1 interaction exerts inhibitory effects that limit effector cells response, prevent the triggering of immune-mediated tissue damage, and regulate the balance between T cell activation and tolerance^25^. A number of studies have indicated that PD-1/PD-L1 pathway is a crucial modulator of host immune responses in regulation of autoimmunity, tumor immunity, transplantation immunity, and allergy^26^,^27^,^28^. The majority of studies have focused on PD-L1 expressed on DCs or macrophages that suppress effector CD4^+^ T cells^26^,^29^. In the present study, we analyzed the expression of PD-L1 on CD19^+^ CD24^+^ CD38^+^ Bregs in breast tumor patients with and healthy individuals. We confirmed that PD-L1 was higher on CD19^+^CD24^+^CD38^+^ Bregs in IBCa patients compared with patients with benign tumor or healthy individuals. Though the percentages of CD19^+^ B cells, CD19^+^CD24^+^CD38^+^ B cells, and CD19^+^ CD24^+^ CD38^+^ PD-L1^+^ B cells were not significantly different in terms of tumor grades, lymph node metastasis, ER, PR, and HER2 status, a tight correlation was seen between upregulated expression of PD-L1 on CD19^+^CD24^+^CD38^+^ Bregs and higher TNM phases of IBCa. More importantly, a tight correlation exists between CD19^+^ CD24^+^ CD38^+^ Bregs and PD-L1 expression in CD19^+^ CD24^+^ CD38^+^ Bregs, and PD-L1 maybe an important molecule in CD19^+^ CD24^+^ CD38^+^ Bregs. We further found that both PD-L1^hi^ and PD-L1^lo^ was significantly higher on CD19^+^ CD24^+^ CD38^+^ Bregs compared with that on non-Bregs (CD19^+^ CD24^−^ CD38^+^ B cells). We therefore speculate that high PD-L1 expression may contribute to the immunosuppressive role of CD19^+^ CD24^+^ CD38^+^ Bregs.

When the expression pattern of PD-1 on CD4^+^ CD25^+^ CD127^−^ Tregs as well as CD4^+^ non-Tregs was examined, we found that PD-1^hi^ was mainly expressed on non-Tregs, and the expression level of PD-1^lo^ was not significantly different between Tregs and non-Tregs. The presence of PD-L1 on CD19^+^ CD24^+^ CD38^+^ Bregs was found to be positively correlated with CD4^+^ CD25^+^ CD127^−^ Tregs, and inversely associated with PD-1^hi^ non-Tregs. As elegantly described by Adnan *et al.*^30^, PD-L1^hi^ B cells could suppress inflammation in EAE model through limiting the expansion of CD4^+^ CXCR5^+^ PD-1^+^ follicular helper T cells (T_FH_-cell). This, in conjunction with our recent finding that that CD19^+^ B cells from IBCa could promoted Tregs in a T and B cells co-culture system^12^, led us to hypothesize that PD-L1 expressed on Bregs might inhibit the proliferation of PD-1^hi^ non-Tregs, and promote the expansion of Tregs to suppress immune response.

As negative function of B cells was demonstrated, B-cell depletion by anti-CD20 antibody was used to augment immunotherapy in multiple solid tumor models^31^. Further investigations confirmed that the absence of B lymphocytes reduced the number and function of Tregs and enhanced the anti-tumor response in a murine tumor model^31^. However, as important immune cells, depletion of B lymphocytes would lead to deficient adaptive immune responses. Furthermore, published studies have showed that the depletion of B lymphocytes by anti-CD20 antibody would enrich CD20^low/−^ Bregs and promote cancer escape^32^. So, Bregs maybe a useful target cells in tumor immunotherapy base on Breg-mediated immunosuppression. And the precise phenotype of Bregs need identified and further investigated in cancer. Here, we have shown the level of PD-L1 was higher on CD19^+^CD24^+^CD38^+^ Bregs in IBCa, and the percentage of PD-L1^+^CD19^+^CD24^+^CD38^+^ Bregs was positively correlated with Tregs, but negatively correlated with PD-1^hi^ non-Tregs. These observations suggested that PD-L1 maybe another important molecule on Bregs, and a critical molecule mediated the promotion of Tregs in advanced breast cancer. CD19^+^ CD24^+^ CD38^+^ PD-L1^+^ Bregs may serve as a target in immunotherapy.

## Materials and Methods

### Reagents

The following monoclonal antibodies and reagents were used in this study: anti-human CD19-ECD (clone: J3-119) (Beckman Coulter Company, Marseille, France) and anti-human CD4-ECD (clone: SFCI12T411) were purchased from Beckman (USA); anti-human CD24-PE-cy7 (clone: eBioSN3), anti-human CD38-APC (clone: HIT2), and anti-human CD25-PE-cy7 (clone: BC96) were from eBiosciencs (San Jose, CA, USA); anti-human CD127-FITC (clone: A019D5) and anti-human PD-1-PE (clone: PD1.3.1.3) were from Miltenyi Biotec (Germany); PDL1-PE (clone: 29E.2A3) was obtained from BioLegend (USA). Fixation/Permeabilization Kit (with GolgiStop protein transport inhibitor containing monensin) was purchased from BD Company (BD Biosciences, USA). The Isotype control antibodies including FITC Mouse IgG1 (clone: MOPC-21), PE-cy7 Mouse IgG1 (clone: MOPC-21), APC Mouse IgG1 (clone: MOPC-21), PE Mouse IgG2b (clone: MPC-11) were purchased from Biolegend (USA). ECD Mouse IgG1 (clone: A07797) was purchased from Beckman (USA). The following antibodies for compensation controls: CD4-FITC (clone: VIT4, Miltenyi Biotec, Germany), CD4-PE (clone: VIT4, Miltenyi Biotec, Germany), CD4-ECD (clone: SFCI12T411, Bechman, USA), CD4-APC (clone: RPA-T4, eBiosciencs, USA), CD4-PE-cy7 (clone: RPA-T4, eBiosciencs, USA). The following antibodies for Fluorescence Minus One (FMO): CD3-FITC (clone: BW264/56, Miltenyi Biotec, Germany), PD-L1-PE (clone: PD1.3.1.3), CD19-ECD (clone: J3-119), CD4-APC (clone: RPA-T4), CD14-PE 770 (clone: TUK4, Miltenyi Biotec, Germany).

Lipopolysaccharide, PMA and calcium-ionomycin were obtained from Sigma (St Louis, USA).

### Patients and peripheral blood cells collection

A total of 153 participants including 98 IBCa, 20 FIBma, and 35 healthy individuals were enrolled in this study. The FIBma patients aged 17–58 years, with a mean of 39.8 years. The healthy individuals aged 25–64 years, with a mean of 45.7 years. The age range of the IBCa patients was from 31 to 90 years, with a mean of 54.2 years. None of the patients with IBCa had received any kind of chemical or radiation therapy before surgery. The characteristics of the IBCa patients were presented in Table 1. 14 out of 98 (14.3%) IBCa were graded as well-differentiated, 63/98 (64.3%) were moderately-differentiated and the rest (21/98, 21.4%) were poorly-differentiated. Carcinoma specimens were classified as stage T1 (24/98, 24.5%), T2 (41/98, 41.8%), T3 (24/98, 24.5%) and T4 (9/98, 9.2%). In addition, local lymph node metastasis occurred in 50 cases of 98 cases (51.0%). Peripheral blood cells were collected from IBCa and FIBma patients, and healthy individuals for flow cytometry analysis of cell surface markers. Written informed consent was obtained from each individual participant. The study protocol was performed in accordance with the guidelines outlined in the Declaration of Helsinki and was approved by the Ethics Committee of Soochow University.

### Peripheral immune cell isolation

Peripheral blood mononuclear cells (PBMCs) were isolated from blood sample via a ficoll density gradient (Amersham Biosciences, Sweden) centrifugation at 1800 rpm for 20 min at 4 °C. PBMCs were washed with FACS buffer and centrifuged at 1800 rpm for 5min prior to labeling with antibodies for flow cytometry analysis.

### Surface staining and flow cytometry

Following isolation, the PBMCs were immediately labelled with the specific fluorochrome-conjugated antibodies including CD4-ECD, CD25-PE-cy7, CD127-FITC and PD-1-PE to identify the surface molecules of Tregs for 30 min at 4 °C. Labeled cells were re-suspended in 0.5 mL cell staining buffer, and analyzed using a flow cytometer (CytomicsTM FC500, Beckman Coulter, USA). The data was analyzed using FlowJo software (version 7.6.2, Ashland, OR, USA). Isotype controls were included for each staining. FMO controls were included for assessing background fluorescence. Meanwhile, CD4-FITC, CD4-PE, CD4-ECD and CD4-PE-cy7 were used for compensation. The compensation and FMO controls were performed as shown in Supplementary Figures 2 and 3.

The surface molecules of Breg were analyzed similarly. In brief, PBMCs were incubated with the specific fluorochrome-conjugated antibodies including CD19-ECD, CD38-APC, CD24-PE-cy7 and PD-L1-PE for 30 min at 4 °C. Labeled cells were re-suspended in 0.5 mL cell staining buffer, and analyzed using flow cytometer with FlowJo software (version 7.6.2, Ashland, OR, USA). Isotype controls were used for each staining, and FMO controls were included to assess background fluorescence. Meanwhile, CD4-PE, CD4-ECD, CD4-APC, and CD4-PE-cy7 were used for compensation.

### Intracellular staining of IL-10 and flow cytometry analysis

Intracellular IL-10 staining and analysis by flow cytometry was performed as previously described^12^. Briefly, PBMCs cells were cultured in the RMPI 1640 media containing 10% fetal calf serum, 200 μg/mL penicillin, 200 μg/mL streptomycin and stimulated with LPS (1 μg/mL) for 72 hour, then stimulated with PMA (50 ng/mL) and ionomycin (750 ng/mL) for 6 hour. Four hours prior to the termination, cells were blocked with GolgiStop (BD Biosciences, USA). Bregs were labeled as described above using CD19-ECD, CD24-Pecy7 and CD38-APC. For intracellular staining, cells were fixed with Fixation/Permeabilization solution and incubated with anti-human IL-10-FITC antibody for 30 min in the dark. Labeled cells were re-suspended in 0.5 mL cell staining buffer, and analyzed with flow cytometry. Isotype controls and FMO controls were included for all experiments. Meanwhile, CD4-FITC, CD4-PE, CD4-ECD, and CD4-APC, were used for compensation.

### Statistical analysis

All of the data were presented as mean ± standard error of the mean and analyzed using ANOVA-test or Student *t* test. ANOVA-test and Student-Newman-Keuls (SNK) for post hoc test were used to compare the mean of the percentage of CD4^+^ T cells, CD19^+^ B cells and their subsets in patients, healthy individuals. Correlation was determined by the Pearson correlation. The *P* < 0.05 and *P* < 0.01 were considered as statistically significant and very significant, respectively. All analyses were done using SPSS 17.0 software (USA).

## Additional Information

**How to cite this article**: Guan, H. *et al.* PD-L1 is a critical mediator of regulatory B cells and T cells in invasive breast cancer. *Sci. Rep.* **6**, 35651; doi: 10.1038/srep35651 (2016).

## Supplementary Material

Supplementary Information

Supplementary Figure 1

Supplementary Figure 2

Supplementary Figure 3

## Figures and Tables

**Table 1:** Relationship between the proportion of CD4^+^ T cells and its subset in PBMCs and the clinicopathological parameters of IBCa patients.

Variables	All cases	T cells		
		CD4^+^	CD4^+^CD25^+^CD127^−^	CD4^+^CD25^+^CD127^−^PD-1^+^
IDCa	98			
Grade	98			
G1	14	24.08 ± 9.13	5.54 ± 2.09	53.49 ± 13.66
G2	63	20.20 ± 8.97	6.03 ± 2.25	45.99 ± 20.93
G3	21	18.90 ± 7.57	6.57 ± 1.99	50.60 ± 12.91
***P***		0.213	0.381	0.314
LN metastasis				
No	48	21.68 ± 16.69	5.90 ± 2.12	47.27 ± 17.93
Yes	50	19.84 ± 8.75	6.20 ± 2.23	48.90 ± 19.37
***P***		0.303	0.496	0.671
TNM				
I	24	23.29 ± 8.60	5.86 ± 2.45	43.44 ± 16.71
II	41	19.61 ± 9.10	5.84 ± 1.70	51.00 ± 20.57
III	24	20.81 ± 7.58	6.45 ± 2.51	47.29 ± 15.61
IV	9	18.91 ± 11.22	6.46 ± 2.55	50.15 ± 21.97
***P***		0.388	0.650	0.462
ER				
Negative	32	21.95 ± 9.38	6.00 ± 1.77	48.91 ± 17.99
Positive	66	20.15 ± 8.58	6.08 ± 2.35	47.72 ± 19.01
***P***		0.348	0.856	0.771
PR				
Negative	56	21.63 ± 9.08	6.01 ± 1.85	47.06 ± 18.80
Positive	42	19.55 ± 8.48	6.12 ± 2.56	49.50 ± 18.47
***P***		0.252	0.793	0.529
HER2				
Negative	62	21.12 ± 8.87	6.10 ± 2.41	49.35 ± 16.97
Positive	36	20.09 ± 8.87	5.98 ± 1.70	45.93 ± 21.26
***P***		0.580	0.796	0.389
Age				
<49 y	39	18.57 ± 8.66	6.23 ± 2.21	47.79 ± 18.32
≥49 y	59	22.18 ± 8.74	5.94 ± 2.16	48.31 ± 18.94
***P***		0.047	0.510	0.894
Tumor size				
<3 cm	59	19.96 ± 8.83	5.89 ± 2.02	46.66 ± 18.56
≥3 cm	39	21.93 ± 8.85	6.31 ± 2.38	50.21 ± 18.69
***P***		0.283	0.346	0.361

**Table 2:** Relationship between the proportion of CD19^+^ B cells and its subset in PBMCs and the clinicopathological parameters of IBCa patients.

Variables	All cases	B cells		
		CD19^+^	CD19^+^CD24^+^CD38^+^	CD19^+^CD24^+^CD38^+^PD-L1^+^
IDCa	98			
Grade				
G1	14	5.32 ± 3.21	9.78 ± 5.41	82.88 ± 20.54
G2	63	5.17 ± 3.15	11.83 ± 6.18	83.09 ± 21.06
G3	21	5.26 ± 3.18	9.50 ± 3.69	86.69 ± 12.63
***P***		0.985	0.178	0.751
LN metastasis				
No	48	5.33 ± 3.60	11.60 ± 6.84	81.30 ± 21.63
Yes	50	5.28 ± 2.85	10.40 ± 4.34	86.08 ± 16.69
***P***		0.931	0.303	0.223
TNM				
I	24	4.74 ± 2.23	10.61 ± 5.69	87.30 ± 9.37
II	41	5.49 ± 3.92	11.07 ± 6.56	76.53 ± 26.35
III	24	5.79 ± 3.00	11.48 ± 4.67	88.30 ± 9.73
IV	9	4.85 ± 2.72	10.31 ± 4.66	94.88 ± 4.72
***P***		0.700	0.939	0.011
ER				
Negative	32	6.36 ± 4.14	11.23 ± 6.98	82.28 ± 20.35
Positive	66	4.79 ± 2.56	10.87 ± 5.03	84.44 ± 18.92
***P***		0.056	0.773	0.605
PR				
Negative	56	5.39 ± 3.61	10.67 ± 6.17	82.79 ± 20.31
Positive	42	5.19 ± 2.66	11.41 ± 5.06	84.99 ± 18.08
***P***		0.759	0.531	0.580
HER2				
Negative	62	5.54 ± 3.17	10.69 ± 6.12	83.61 ± 17.05
Positive	36	4.90 ± 3.32	11.50 ± 4.95	83.96 ± 22.97
***P***		0.345	0.501	0.930
Age				
<49 y	39	5.30 ± 3.72	12.13 ± 6.84	82.25 ± 23.48
≥49 y	59	5.31 ± 2.89	10.23 ± 4.73	84.72 ± 16.14
***P***		0.983	0.108	0.539
Tumor size				
<3 cm	59	4.69 ± 3.03	11.09 ± 5.17	85.48 ± 18.81
≥3 cm	39	6.24 ± 3.33	10.83 ± 6.50	81.21 ± 20.04
***P***		0.019	0.829	0.295
