# Supplementary material for: PD-L1 is a critical mediator of regulatory B cells and T cells in invasive breast cancer
Source: Sci Rep. 2016 Oct 20;6:35651. doi: 10.1038/srep35651 (PMC5071845; doi:10.1038/srep35651)
Supplement: Supplementary Information [file srep35651-s1.doc]

**Title page**

**PD-L1 is a critical mediator of regulatory B cells and T cells in invasive breast cancer**

**Honggeng Guan^1,2*^, Yuqiu Wan^2,*^, Jing Lan^2,*^, Qin Wang^3^, Zhangyu Wang^2^, Yecheng Li^4^, Jiqing Zheng^1^, Xueguang Zhang^5^, Zemin Wang^6^, Yueping Shen^7^, Fang Xie^1^**

**Supplementary Figure legends**

**Supplementary Figure 1. The pattern of PD-1 on CD4^+^ T lymphocyte in PBMCs of IBCa patients**

The pattern of PD-1 on CD4^+^CD25^+^CD127^low/-^ Tregs, CD4^+^CD25^+^CD127^+^ T cells, and CD4^+^CD25^-^ T cells in PBMCs of IBCa patients was analyzed by cytometry.

**Supplementary Figure 2. The compensation was performed in experiment design and data analysis**

PBMCs were collected and incubated with CD4-FITC, CD4-PE, CD4-ECD, CD4-APC, and CD4-PE-cy7 respectively, and analyzed by cytometry for compensation. Isotype controls were used for each staining.

**Supplementary Figure 3. Fluorescence Minus One (FMO) control for PE was performed in experiment design and data analysis**

PBMCs were collected and incubated with CD3-FITC, CD4-ECD, CD14 PE-770 and PE Mouse IgG1 respectively, and analyzed by flow cytometry.
