# Supplementary figures and images for: PD-L1 is a critical mediator of regulatory B cells and T cells in invasive breast cancer
Source: Sci Rep. 2016 Oct 20;6:35651. doi: 10.1038/srep35651 (PMC5071845; doi:10.1038/srep35651)

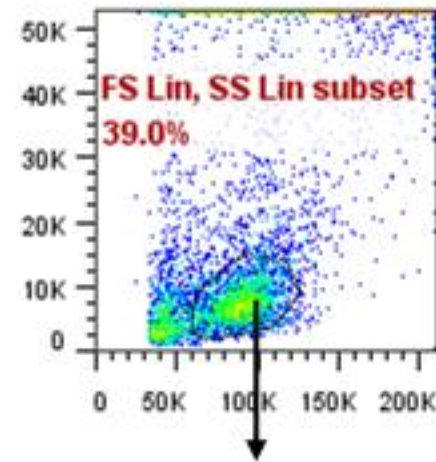

Gated to SSCD4<sup>+</sup>

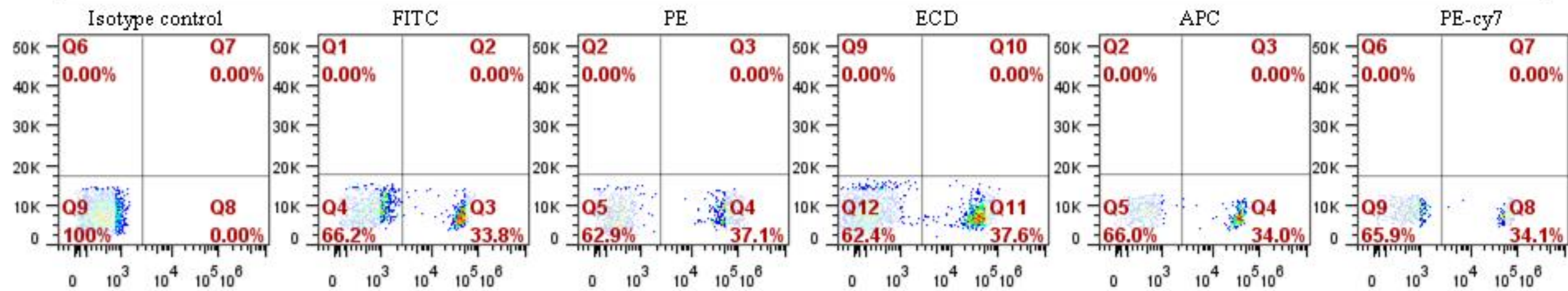

Supplement: Supplementary Figure 2 [file srep35651-s3.pdf]

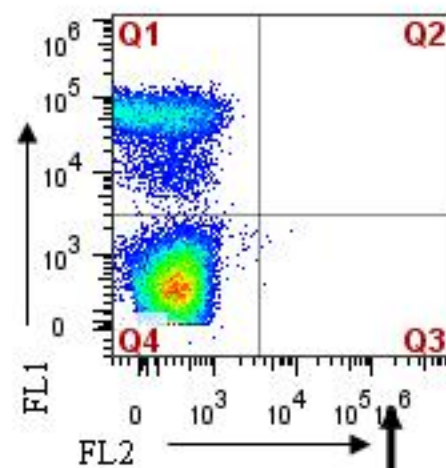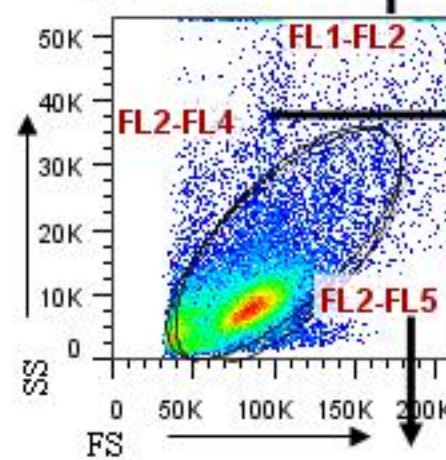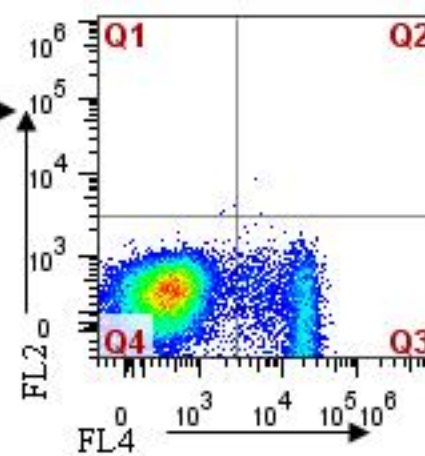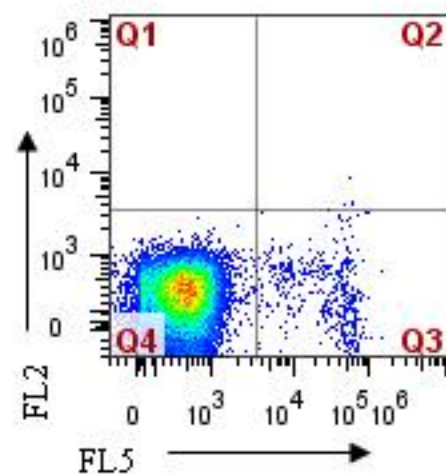

Supplement: Supplementary Figure 3 [file srep35651-s4.pdf]
