# Supplementary material for: PD-L1 is a critical mediator of regulatory B cells and T cells in invasive breast cancer
Source: Sci Rep. 2016 Oct 20;6:35651. doi: 10.1038/srep35651 (PMC5071845; doi:10.1038/srep35651)
Supplement: Supplementary Figure 1 [file srep35651-s2.pdf]

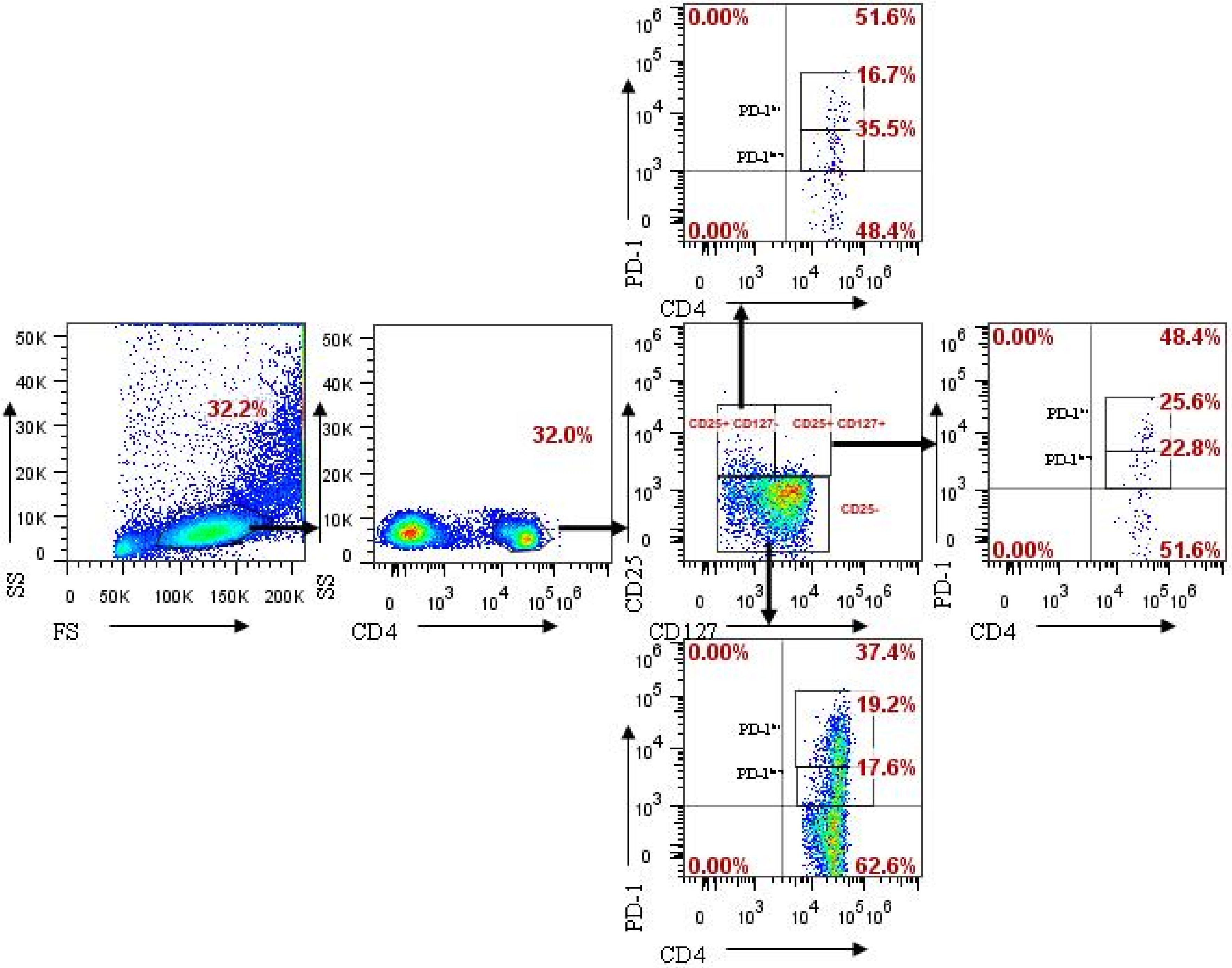

SS
FS
0
50K
100K
150K
200K
10K
20K
30K
40K
50K
32.2%
CD4
32.0%
CD25
CD127
CD25+ CD127-
CD25+ CD127+
CD25-
PD-1
0.00%
51.6%
16.7%
35.5%
48.4%
25.6%
22.8%
37.4%
19.2%
17.6%
62.6%
